# Introducing transcatheter aortic valve implantation with a new generation prosthesis: Institutional learning curve and effects on acute outcomes

**DOI:** 10.1007/s12471-016-0925-4

**Published:** 2016-12-05

**Authors:** G. D’Ancona, H. U. Agma, S. Kische, G. El-Achkar, M. Dißmann, J. Ortak, H. Ince, U. Ketterer, A. Bärisch, A. Öner

**Affiliations:** 1 Department of Cardiology, Vivantes Klinikum im Friedrichshain und Am Urban, Berlin, Germany; 2 Rostock University Medical Center, Rostock, Germany; 3 Department of Cardiology, Vivantes Humboldt-Klinikum, Berlin, Germany

**Keywords:** Trans-catheter aortic valve implantation, Percutaneous, Learning curve

## Abstract

**Objectives:**

We present our single-centre experience with the direct flow medical (DFM) trans-catheter aortic valve implantation (TAVI) prosthesis addressing the impact of learning curve upon outcomes.

**Background:**

The DFM has been recently introduced for TAVI. The prosthesis presents original design and implantation features.

**Methods:**

Patients were divided into three groups according to the chronological implantation sequence that reflected technical skills acquisition of the entire team.

**Results:**

Group I included the first 20 patients (early learning phase), group II the second 20 patients (proctoring to other members of the team), and group III the following 93 patients (technique consolidation). Differences in baseline and procedural variables were analysed. Nonparametric correlation and linear regression were used to identify changes according to institutional cumulative experience. There was a significant correlation between catheterisation time and institutional experience (rho = −0.4; *p* < 0.0001) confirmed at linear regression (beta = −0.2; *p* = 0.001; CI: −0.3 – −0.08). Moreover, there was lower rate of valve retrieval in group III (15% vs. 20% vs. 10%; *p* = 0.5). No intra-procedural mortality was reported and improved early safety (at 30 days) was observed (80% vs. 85% vs. 87.1; *p* = 0.7). At hospital discharge, valve haemodynamic performance was satisfactory with only mild regurgitation in 10% (I), 20% (II), and 9.7% (III) (*p* = 0.8).

**Conclusions:**

DFM adequate sizing and implantation can be achieved after the early learning phases. A significant reduction in catheterisation time is reported after the first 20 patients. Results remain satisfactory during the proctoring and technical consolidation phase.

## Introduction

In the present manuscript we summarise our experience with a recently introduced inflatable and fully retrievable metal-free transcatheter aortic valve implantation (TAVI) prosthesis (Direct Flow Medical, DFM, Santa Rosa, California, USA) focusing on our institutional learning curve and its impact on patient selection, intraoperative variables, and postoperative outcomes.

## Methods

Perioperative data were prospectively collected in a series of patients undergoing TAVI with the DFM prosthesis. Patients had previously signed informed consent to the procedure and to clinical data handling for scientific purposes. Data were reported according to the Valve Academic Research Consortium definitions (VARC-2) [1].

The design features of this bovine pericardial prosthesis have already been described in the literature [2, 3]. The prosthesis includes three pericardial leaflets mounted on an inflatable flexible metal-free frame formed by a lower and upper ring connected by lateral struts. Three hollow wires for valve pressurisation and fine-tuned positioning are screwed to the upper ring. The valve is mounted on a flexible 18-F delivery system.

All patients included in this series had been diagnosed with severe symptomatic aortic valve stenosis. Valve sizing was performed on the basis of the preoperative cardiac computed tomography (CT) imaging. Pure aortic insufficiency, previous aortic valve surgery (replacement/repair), and previous TAVI were excluded from the present analysis.

To assess the learning process and its impact upon patient selection, intra-procedural results and hospital outcomes, we divided the entire cohort of patients treated by our TAVI team with the DFM into three groups, according to the chronological sequence of implantation, which reflects device amelioration and technical skills acquisition/evolution for the entire TAVI team. Group I included the first 20 patients and was defined as the early learning phase. Only two operators in our team were involved in this phase and, at that point, only two valve sizes were available (25 and 27 mm) and an early version of the DFM delivery catheter was used. Group II included the second 20 patients and was defined as the proctoring to other members of the team phase. At that stage new members of the team (in total two new members) were introduced to the DFM system, an implemented delivery catheter was produced, and an extra size for the DFM prosthesis was available (23 mm). Group III included the remaining and following 93 patients and was defined as the phase of technique consolidation and improvement.

The effect of the learning curve was assessed by procedure time efficiency (operating time, catheterisation time from valve sheath introduction in the femoral artery to its removal and fluoroscopy time) and by outcomes (intra-procedural and 30-day).

Differences between the three groups were tested by means of one-way analysis of the variance (ANOVA) followed by post-hoc Tukey test (to define differences in between groups), and chi-square and Fisher’s exact tests whenever appropriate. Nonparametric correlation (Spearman rho) and linear regression were used to identify changes according to institutional cumulative experience. The statistical calculations were run using the SPSS (version 22) software.

## Results

A total of 133 patients with symptomatic severe aortic valve stenosis were treated; the first 20 were included in group I, the second 20 in group II, and the following 93 in group III. Table 1 summarises the preoperative data in the three groups. Intra-procedural findings are summarised in Table 2.

**Table 1:** Demographic, clinical and anatomic characteristics of patients

	Group I (20)	Group II (20)	Group III (93)	*p-value*
Age (years)	80.3 ± 5.9	79.6 ± 5.2	80.9 ± 5.4	0.6
Gender-Female	40.0%	50.0%	35.5%	0.5
BMI	28.0 ± 5.1	28.1 ± 4.5	28.1 ± 10.9	1.0
Log. euroSCORE	22.9 ± 12.5* (20.1; 6.6–44.0)	12.4 ± 5.4 *, ** (10.9; 5.5–23.6)	19.5 ± 12.8** (16.6; 5.8–84.2)	0.02
EuroSCORE II	5.8 ± 6.8 (3.4; 1.9–31.5)	4.2 ± 4.0 (2.9; 1.2–18.1)	6.4 ± 6.7 (4.1; 1.3–47.8)	0.4
STS Score(M)	5.2 ± 2.6 (4.7; 1.4–13.4)	4.1 ± 2.0 (3.9; 1.8–10.3)	4.8 ± 3.2 (3.8; 1.3–21.2)	0.5
*Pre-procedural TTE*				
LVEF (%)	49.4 ± 13.4	57.3 ± 12.4	52.5 ± 14.5	0.02
AV gradient mean (mm Hg)	42.5 ± 13.7	49.8 ± 12.9***	40.7 ± 14.2***	0.04
AVA (cm^2^)	0.71 ± 0.1	0.65 ± 0.1	0.74 ± 0.1	0.4
*Cardiac CT*				
LVOT eccentricity index	0.26 ± 0.08	0.29 ± 0.08	0.28 ± 0.08	0.3
LVOT perimeter derived diameter (mm)	24.9 ± 1.6	24.0 ± 1.4	25.2 ± 2.5	0.07
Annulus aortic eccentricity index	0.20 ± 0.09	0.23 ± 0.09	0.22 ± 0.08	0.3
Annulus perimeter derived diameter (mm)	24.7 ± 1.5	24.1 ± 1.4	25.2 ± 2.2	0.07
Calcification total extension (mm)	14.5 ± 4.2****	12.3 ± 3.5	11.5 ± 3.6****	0.005

*p < 0.05; ** p < 0.05; *** p < 0.05; **** p < 0.005

*BMI* body mass index; *Log. EuroSCORE* Logistic EuroSCORE; *STS-Score(M)* Society of Thoracic Surgeons Mortality Risk Score; *TEE* transoesophageal echocardiography; *AV-gradient mean* mean aortic transvalvular gradient; *AVA* aortic valve area; *CT* computerised tomography; *LVOT* left ventricular outflow tract; *Eccentricity Index* ((Diameter max-Diameter min)/(Diameter max)); calcification total extension, from LVOT towards aortic bulbus

**Table 2:** Procedural, morbidity, and mortality data

	Group I (20)	Group II (20)	Group III (93)	*p-value*
Procedure time, min	110.1 ± 42.3*	96.8 ± 35.9	90.8 ± 30.8*	0.08
Catheter time, min	56.9 ± 38.7**^,^ ***	35.1 ± 11.8**	34.0 ± 22.5***	0.001
Contrast, ml	99.0 ± 51.7^a^	134.3 ± 47.8	152.8 ± 110.5^a^	0.08
Radiation time, min	23.5 ± 13.2	27.4 ± 16.3	22.9 ± 11.4	0.4
Radiation dose-area product, µGcm^2^	10303.9 ± 6193.3	13083.9 ± 9830.8	13202.3 ± 11857.6	0.6
*Access management*				0.2
Percutaneous	100.0%	95.0%	87.1%	
Cut-down	0.0%	5.0%	12.9%	
Implanted prosthesis size-average, mm	26.1 ± 1.0	25.5 ± 1.1	26.1 ± 1.7	0.2
DF 23	–	5.0%	8.6%	
DF 25	45.0%	65%	39.8%	
DF 27	55.0%	25.0%	33.3%	
DF 29	–	–	15.1%	
CV 26	–	5.0%	1.1%	
CV 31	–	–	2.2%	
Predilatation balloon size, mm	24.3 ± 1.0^||, #^	22.3 ± 1.0^||^	22.7 ± 3.0^#^	0.03
Retrieval frequency Pull through	15.0% (3) 15.0% (3)	20.0% (4) 15.0% (3)	10.8% (10) 7.5% (7)	0.4 0.4
*Access closure*				0.002
Percutaneous	75.0%	70.0%	79.6%	
Stent	25.0%	15.0%	2.2%	
Hospitalisation after procedure, days	14.0 ± 10.9^+, ©^	8.3 ± 2.8^+^	9.1 ± 5.9^©^	0.009
All-cause mortality	10.0%	10.0%	6.5%	0.7
Cardiovascular	5.0%	10.0%	3.2%	
Non-cardiovascular	5.0%	0.0%	3.2%	
Myocardial infarction	5.0%	0.0%	0.0%	0.3
Stroke	5.0%	0.0%	3.2%	0.5
Life-threatening or disabling bleeding	5.0%	0.0%	1.1%	0.03
Major bleeding	15.0%	5.0%	2.2%	0.03
Major vascular complication	0.0%	5.0%	1.1%	0.05
New pacemaker	25.0%	15.0%	13.0%	0.6
Device success^b^	90.0%	75.0%	90.3%	0.6
Early safety (at 30 days)^b^	80.0%	85.0%	87.1%	0.7

*p = 0.07; **p < 0.05; ***p < 0.05; ^a^ *p* = 0.07; ^||^ *p* < 0.05; ^#^ *p* < 0.05; ^+^ *p* < 0.05; ^©^ *p* < 0.05

^b^As defined by VARC criteria

**Fig. 1:**
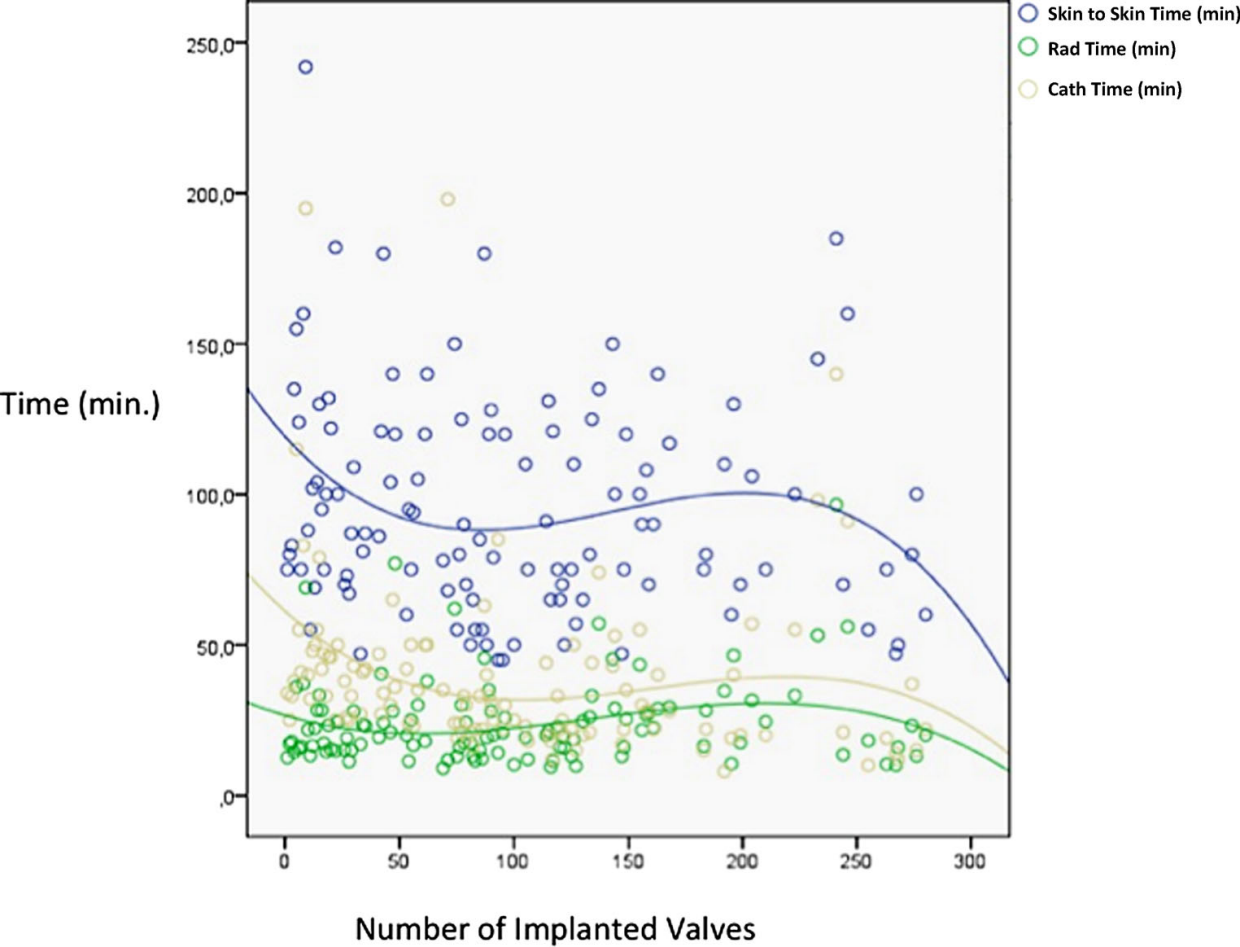
Correlation between catheterisation time, procedural time, radiation time (*Y-axis*) and institutional experience (*Y-axis*: minutes and *X-axis*: numbers of implanted valves)

With experience there was a reduction in procedural and radiation time with a positive trend in the total use of contrast (Table 2). Catheterisation time (the time elapsing from introduction to removal of the valve delivery system) was significantly reduced from group I to group III (*p* = 0.001). In addition, there was a significant correlation between catheterisation time and institutional experience (rho = −0.4; *p* < 0.0001) (Fig. 1), which was confirmed at linear regression (beta = −0.2; *p* = 0.001; CI: −0.3 – −0.08).

When looking at the overall DFM retrieval and pull through rate, this was not significantly lower in group III (*p* = 0.4). In four cases (1 in group II and 3 in group III) Medtronic CoreValves (Minneapolis, Minnesota, USA) were implanted after having uneventful retrievals of the DFM prostheses. In one case the DFM catheter was kinked and fractured twice as a result of a very tortuous and calcified iliac-femoral access. In a second case the DFM could not be inflated within a small and hypertrophic left ventricle, possibly as a result of entanglement with the mitral sub-valvular apparatus. In two other cases with large aortic annuli (29 mm) and limited aortic calcifications, 31 mm Medtronic CoreValves were implanted after failed attempts at implanting the largest DFM (29 mm) prosthesis. No patients required emergent thoracotomy and/or conversion to conventional aortic valve replacement.

Total hospitalisation was significantly reduced throughout the experience (*p* = 0.009) and lower (non-significant) total and cardiovascular in-hospital mortality was reported in group III (Table 2). No mortality was directly related to the DFM prosthesis. Apart from one case, all ten deceased patients had a very complex preoperative comorbid profile including frailty and advanced age (81–94 years) in seven patients, depressed LVEF <30% in three patients, and dialysis in one patient. Vascular bleeding occurred significantly more often in the early phases of the experience (*p* = 0.03).

There was no statistically significant difference in device success and early safety according to VARC criteria [1] in the three groups. There was a higher device success rate in the very early and last phases. Moreover, a higher early safety was reported in the last phase of our learning curve (Table 2).

Pre-discharge echocardiography data are summarised in Table 3. Although higher (non-significant) trans-prosthetic gradients were observed in the late phase, mild prosthetic aortic valve stenosis [1] (mean prosthetic gradient between 20–40 mm Hg and peak velocity between 3–4 m/s) occurred at discharge in 2 patients in group I (10%), 4 patients in group II (21.1%), and in 6 patients in group III (6.7%) (*p* = 0.6).

**Table 3:** Postoperative echocardiographic data (through 30 days)

	Group I (20)	Group II (20)	Group III (93)	*p-value*
LVEF (%)	52.8 ± 11.4	59.6 ± 16.1	54.7 ± 14.1	0.4
Max gradient, mm Hg	27.4 ± 11.7	26.8 ± 8.8	28.8 ± 12.8	0.8
Mean gradient, mm Hg	12.7 ± 6.5	14.6 ± 6.5	16.1 ± 7.0	0.2
Peak velocity, m/s	2.8 ± 0.6	2.5 ± 0.7	2.7 ± 0.6	0.5
*Transvalvular (central) regurgitation*				0.6
None	90.0%	80.0%	88.2%	
Mild	10.0%	20.0%	11.8%	
*Paravalvular regurgitation*				0.4
None	90.0%	80.0%	90.3%	
Mild	10.0%	20.0%	9.7%	
*Total aortic regurgitation*				0.8
None	90.0%	80.0%	84.9%	
Mild	10.0%	20.0%	15.1%	

## Discussion

This metal-free, inflatable, fully retrievable, and repositionable valve offers some original and potentially advantageous features which, even within the premises of centres with recognised wide experience with other TAVI devices, can be appreciated only through an initial learning phase.

In a recent prospective multicentre trial of the DFM, a preplanned roll-in cohort of three patients per site was accepted as the necessary number to allow the operator to gain technical expertise with this new technology [3]. The 20-patient limit we have proposed to define the first and second phases of learning reflects what happened in our practice and in the skills acquisition process within our facility.

We would like to remark on the importance of distinguishing two different stages in the learning process: first the patient selection stage and second the implantation (technical) stage. As new prostheses are introduced into the market, operators should be prepared to treat aortic stenosis using a patient-tailored approach that should be based upon the peculiar valve design and the different anatomical scenarios that may be encountered. In fact, during the same period we treated over 100 patients with other TAVI prostheses.

In particular, extension and distribution of aortic calcification has played an important role in guiding prosthesis selection. With time we have developed a structured selection algorithm that takes the DFM design and the specific characteristics of the patient’s aortic unit into consideration. The DFM has a minimised traumatic effect on the native tissues, as a result of its metal-free structure and contained radial force. The valve design includes an annulus to aortic ring height of 15 mm (for the 23, 25, 27 mm valves) to 16 mm (for the 29 mm valve) and ideally the prosthesis height should overcome and ‘embrace’ the total vertical extension (across the annulus of the aortic valve) of the calcification to allow complete and symmetric expansion of the upper ring of the prosthesis. Moreover, having a landing zone for the lower ring of the prosthesis that is free from calcifications will enhance sealing and minimise the risk of paravalvular leak. With this concept in mind, whenever bulky calcifications are extending continuously and uninterruptedly from the aortic annulus into the left ventricular outflow tract (LVOT) for a total depth of >3 mm, we prefer to use a different TAVI prosthesis (with a stronger radial force), to reduce the risk of paravalvular leak.

These concepts are substantiated in our results section where it emerges that, throughout our overall experience, the average calcification vertical extension (from the LVOT to the aortic bulbus) is constantly and significantly reduced from an average of 14.5 mm, in the initial phase, to the most recent value of 11.5 mm. This selection strategy has allowed us to eliminate the occurrence of moderate paravalvular leak and to reduce the rate of mild regurgitation to below 10%.

For what concerns our implantation technique, we have to report a constant evolution. In fact through the three phases of our institutional learning curve we have modified and optimised the implantation technique of this newly introduced prosthesis. In the last phase of our experience we consistently applied a modified technique for DFM implantation that includes an initial ‘low-pressure’ inflation of the valve followed by a patient tailored technique for valve positioning (alternate curve technique), according to the calcification distribution within the aortic unit. Both modifications have been described previously [4].

Optimisation of the DFM implantation technique is reflected in a significant reduction in catheterisation time during the learning process, as confirmed by our correlation and linear regression analysis. The increased use of contrast agent that emerges from our data deserves a comment apart. This finding most probably results from the fact that as experience grew, we dedicated more attention to optimising the final position of the DFM prosthesis and collecting intraoperative imaging for research purposes.

Moreover, as we gained experience with the valve intracardiac navigation we also reduced our prosthesis retrieval and pull-through rates. The 10.8 and 7.5% retrieval and pull-through rates that we observed in the last phase of our experience are in line with those proposed in the prospective trial [3]. We have also started to treat patients with borderline anatomical features that we would have not treated in the very early stages. By testing the possible boundaries of this technology we have at times also incurred failures, even in the last phase of our learning curve. In four cases, this resulted in full retrieval of the DFM and successful implantation of a different device. Although we were perfectly aware of the anticipated difficulties (tortuous iliac-femoral axis, extremely hypertrophic and small ventricle, large and only slightly calcified aortic annulus) in all four cases, we decided to proceed with the DFM implantation. The decision was guided by the fact that certain anatomical features are difficult to approach with any TAVI prostheses and we were aware of the fact that eventual DFM retrieval could have been performed at any time, maintaining perfectly stable haemodynamic conditions and without compromising the final outcome.

A contained paravalvular regurgitation rate has been reported at echocardiography since the beginning of our experience with the DFM valve. Our findings are very encouraging and are in line with those reported in the multicentre trial [3]. In the last part of our experience we reported a post-procedural mean trans-prosthetic gradient value of 16.1 mm Hg, which is slightly higher than the 12.7 mm Hg gradient documented in the initial phase of our experience. Although a mean gradient of 12.6 mm Hg has been reported in the DFM prospective trial, it is not clear if this mean value also included evaluations performed intraoperatively, under general anaesthesia [3]. The increased mean gradient we have reported may have resulted from the introduction of the 23 mm DFM prosthesis. This prosthesis size only became available at the second stage and, in fact, even in the prospective trial no patients were treated with the 23 mm DFM [3]. In addition, other authors have recently documented a discrepancy between invasive and echocardiographic transvalvular gradient measurements after DFM implantation [5].

Moreover, in a recent propensity matched study comparing the DFM prosthesis with the Medtronic CoreValve and Edwards Sapien XT (Edwards Lifesciences, Irvine, California, USA), Zhang et al. demonstrated similar immediate postoperative trans-prosthetic gradients with the three different prostheses [6].

With experience we have witnessed a decrease in the length of hospitalisation, major morbidity, permanent pacemaker implantation rate, and in-hospital mortality. In addition, even in the more recent part of our experience we reported an early safety at 30-days [1] of 87.1%. We are aware that this value is lower than the 91% rate reported in the prospective trial [3]. The difference is mainly due to the higher 30-day mortality rate (6.5% vs. 1.3%) that we experienced. We have to remark that the cardiovascular mortality was 3.2% in group III and that, in the overall experience, none of the deaths were peri-procedural and/or directly related to the prosthesis implantation and to its function. Finally, as already elucidated in the results section, most of the deaths occurred in elderly and comorbid patients.

## Conclusions

Because of its original design and implantation technique, TAVI with the DFM has a learning phase. Although adequate patient selection and valve implantation can be achieved after the early learning phases, results were improved with the evolution of our learning process.
